# Does antibacterial treatment for urinary tract infection contribute to the risk of breast cancer?

**DOI:** 10.1054/bjoc.1999.1047

**Published:** 2000-02-01

**Authors:** P Knekt, H Adlercreutz, H Rissanen, A Aromaa, L Teppo, M Heliövaara

**Affiliations:** National Public Health Institute, Mannerheimintie 166, Helsinki, 00300, Finland; Folkhälsan Research Center, Institute for Preventive Medicine, Nutrition and Cancer, Helsinki, 00300, Finland; Department of Clinical Chemistry, University of Helsinki, Helsinki, 00290, Finland; Finnish Cancer Registry, Helsinki, 00170, Finland

**Keywords:** antibiotics, breast, chemotherapy, longitudinal studies, neoplasms, urinary tract infections

## Abstract

Low lignan status has been reported to be related to an elevated risk of breast cancer. Since lignan status is reduced by antibacterial medications, it is plausible to hypothesize that repeated use of antibiotics may also be a risk factor for breast cancer. History of treatment for urinary tract infection was studied for its prediction of breast cancer among 9461 Finnish women 19–89 years of age and initially cancer-free. During a follow-up in 1973–1991, a total of 157 breast cancer cases were diagnosed. Women reporting previous or present medication for urinary tract infection at baseline showed an elevated breast cancer risk in comparison with other women. The age-adjusted relative risk was 1.34 (95% confidence interval (CI) = 0.98–1.83). The association was concentrated to women under 50 years of age. The relative risk for these women was 1.74 (95% CI 1.13–2.68), whereas it was 0.97 (95% CI 0.59–1.58) for older women. The relative risk in the younger age-group was 1.47 (95% CI 0.73–2.97) during the first 10 years of follow-up, and 1.93 (95% CI 1.11–3.37) for follow-up times longer than 10 years. These data suggest that premenopausal women using long-term medication for urinary tract infections show a possible elevated risk of future breast cancer. The results are, however, still inconclusive and the hypothesis needs to be tested by other studies. © 2000 Cancer ResearchCampaign


British Journal of Cancer (2000) **82**(5), 1107–1110
© 2000 Cancer Research Campaign
DOI: 10.1054/ bjoc.1999.1047, available online at http://www.idealibrary.com on IDEAL®

### Does antibacterial treatment for urinary tract infection contribute to the risk of breast cancer?

**P Knekt[1], H Adlercreutz[2,3], H Rissanen[1], A Aromaa[1], L Teppo[4] and M Heliövaara[1]**

[1]National Public Health Institute, Mannerheimintie 166, 00300 Helsinki, Finland; [2]Folkhälsan Research Center, Institute for Preventive Medicine, Nutrition and Cancer, 00300 Helsinki, Finland; [3]Department of Clinical Chemistry, University of Helsinki, 00290 Helsinki, Finland; [4]Finnish Cancer Registry, 00170 Helsinki, Finland

**Summary** Low lignan status has been reported to be related to an elevated risk of breast cancer. Since lignan status is reduced by antibacterial medications, it is plausible to hypothesize that repeated use of antibiotics may also be a risk factor for breast cancer. History of treatment for urinary tract infection was studied for its prediction of breast cancer among 9461 Finnish women 19–89 years of age and initially cancer-free. During a follow-up in 1973–1991, a total of 157 breast cancer cases were diagnosed. Women reporting previous or present medication for urinary tract infection at baseline showed an elevated breast cancer risk in comparison with other women. The age-adjusted relative risk was 1.34 (95% confidence interval (CI) = 0.98–1.83). The association was concentrated to women under 50 years of age. The relative risk for these women was 1.74 (95% CI 1.13–2.68), whereas it was 0.97 (95% CI 0.59–1.58) for older women. The relative risk in the younger age-group was 1.47 (95% CI 0.73–2.97) during the first 10 years of follow-up, and 1.93 (95% CI 1.11–3.37) for follow-up times longer than 10 years. These data suggest that premenopausal women using long-term medication for urinary tract infections show a possible elevated risk of future breast cancer. The results are, however, still inconclusive and the hypothesis needs to be tested by other studies.
© 2000 Cancer Research Campaign

**Keywords:** antibiotics; breast; chemotherapy; longitudinal studies; neoplasms; urinary tract infections

An adequate level of the lignan enterolactone in the body may provide protection against breast cancer. In accordance with this hypothesis an elevated risk of breast cancer has been reported in women with a low plasma enterolactone (Hulten et al, 1998) and urinary enterolactone level (Adlercreutz et al, 1982; Ingram et al, 1997). Whole-grain products, berries and some vegetables contribute to the enterolactone level in the body (Adlercreutz, 1998).

Enterolactone is produced by microbial transformation in the gut from the plant precursors matairesinol and secoisolariciresinol (Setchell et al, 1981; Borriello et al, 1985). There is a remarkable variation in the ability of human subjects to produce enterolactone from the precursors probably due to differences in bacterial microflora (Adlercreutz and Mazur, 1997). Administration of antibiotics or chemotherapeutics strongly reduced the enterolactone level in body fluids (Setchell et al, 1981; Adlercreutz et al, 1986) and long-term treatment with antibiotics could thus be a risk factor for breast cancer. Bacteriuria is one of the most frequent causes for such treatment in Finland. We have therefore studied the effect of urinary tract infection, bacteriuria and antibacterial treatment on breast cancer risk in the Finnish Mobile Clinic Health Examination Survey cohort of 9461 women during an 18-year follow-up period.

*Received 28 April 1999*
*Revised 5 August 1999*
*Accepted 11 August 1999*

*Correspondence to:* P Knekt

### POPULATION AND METHODS

Between 1973 and 1977, the Finnish Mobile Clinic Health Examination Survey carried out multiphasic health examinations in 12 municipalities in four regions of Finland (Knekt et al, 1998). In each of these four geographic regions, all inhabitants or a random sample of inhabitants of one rural municipality and one urban or semiurban municipality as well as the employees of one factory were invited to attend the examination. A total of 9633 women 19–89 years of age participated in the examination.

All participants completed a premailed questionnaire checked at the baseline examination. The questionnaire yielded information on residence, education, marital status, smoking, number of childbirths, previous diseases and use of medication. Body height and weight were measured at the baseline examination, and the body mass index (weight (kg)/height$^2$ (m$^2$)) was calculated. Previous history of a number of diseases was obtained using specific questions: ‘Have you had, according to a physician’s diagnosis, cystitis, pyelonephritis, urinary tract infection or bacteria in the urine?’ and, if yes: ‘Have you ever received medication for it?’ and ‘Are you taking medication for it at present?’ On the basis of these questions, a category of previous or current antibacterial treatment for urinary tract infection was defined. To study the repeatability of the responses, a sample including 131 women was re-examined after 6 months. The history of medical treatment for urinary tract infection was well repeatable (kappa = 0.72).

In another part of the questionnaire, the participants were asked to give the names of all the medications they had taken in the previous 3 months. The preparations were categorized into the main groups of the Finnish Remedia Fennica of 1974, including 12a: antibiotics proper (penicillins, streptomycin, chloramphenicol, tetracyclines, erythromycin, cephalosporins and their analogues); 12b: sulphonamides; and 12e: various other antimicrobial agents (nitrofurantoin, nalidixate sodium, trimethoprim and its combinations with sulphonamides). Among the 290 women who had reported present use of medication for urinary tract infection, a preparation from group 12b was indicated by 155 (53.4%), 12b or 12e by 282 (97.2%); and 12a, 12b or 12e by 289 (99.7%).

To screen for bacteriuria, all female participants gave midstream urine samples. The presence of urinary bacteria was determined using Uricult[R] culture. Growth of $>10^4$ $ml^{-1}$ was considered positive in the present study. At the 6-month re-examination, 33.5% of the women who had been positive at the baseline screening were found to have definite bacteriuria (growth of $> 10^5$ $ml^{-1}$ of a pathogenic organism), and these women were referred to care.

Information regarding cancer incidence during the follow-up was obtained by linking data in the nationwide Finnish Cancer Registry (Teppo et al, 1994) to the data of the study population. Reporting to the Registry has been obligatory since 1961 and the coverage of the Registry is practically complete. A total of 9461 women were at risk after exclusion of persons who had diagnosed cancer before or during the baseline examination. During the follow-up period from the 1973–1977 baseline examination to late 1991, 157 female breast cancer cases (World Health Organization, 1955) were diagnosed.

The Cox proportional hazards model was used to estimate the association between treatment for urinary tract infection and the incidence of breast cancer (Cox, 1972). Adjustment for potential confounding factors was performed by including these in the models. Interaction terms between antibacterial medication and age were also included. Relative risks between users and nonusers of antibacterial medication were estimated, based on the models.

### RESULTS

Those women who developed breast cancer during the 18-year-long follow-up period were older, more often from urban or industrial areas, had a higher level of education, were leaner and taller, had a smaller number of childbirths and consumed more alcohol than women not developing the disease (Table 1). Although no elevation in the incidence of breast cancer was noted in women who were screening positive for bacteriuria at baseline, those with a previous history of antibacterial treatment for bacteriuria showed a relative risk (RR) for the neoplasm of 1.34 (95% confidence interval (CI) 0.98–1.83) in comparison with other women. The

**Table 1** Relative risk[a] (95% confidence interval) of breast cancer between categories of potential risk factors

| Factor | Category | No. of cases | No. at risk | Relative risk | 95% confidence interval |
|---|---|---|---|---|---|
| Age | <30 | 10 | 2065 | 1 | |
| | 30–39 | 34 | 1830 | 3.76 | 1.86–7.62 |
| | 40–49 | 43 | 1786 | 4.97 | 2.50–9.88 |
| | 50–59 | 27 | 1726 | 3.44 | 1.66–7.10 |
| | 60–69 | 28 | 1361 | 5.49 | 2.67–11.32 |
| | ≥70 | 15 | 693 | 9.06 | 4.05–20.25 |
| Region type | Urban | 85 | 4423 | 1 | |
| | Agriculture | 41 | 3637 | 0.55 | 0.38–0.80 |
| | Industry | 31 | 1401 | 0.96 | 0.63–1.47 |
| Education | Low | 97 | 6253 | 1 | |
| | Intermediate | 45 | 2578 | 1.49 | 1.03–2.16 |
| | High | 15 | 589 | 1.92 | 1.11–3.32 |
| Marital status | Unmarried | 27 | 1712 | 1 | |
| | Married | 101 | 6150 | 0.88 | 0.57–1.34 |
| | Divorced | 24 | 1235 | 0.78 | 0.43–1.42 |
| | Widowed | 5 | 362 | 0.70 | 0.27–1.82 |
| Body mass index | <22.5 | 49 | 3015 | 1 | |
| | 22.5–24.9 | 39 | 2102 | 0.88 | 0.57–1.35 |
| | 25.0–27.4 | 24 | 1689 | 0.59 | 0.36–0.99 |
| | 27.5–29.9 | 21 | 1186 | 0.69 | 0.40–1.89 |
| | ≥30 | 24 | 1468 | 0.61 | 0.36–1.03 |
| Parity | Nulliparous | 40 | 2296 | 1 | |
| | 1–2 | 70 | 3740 | 0.97 | 0.66–1.43 |
| | ≥3 | 47 | 3425 | 0.59 | 0.38–0.90 |
| Smoking | Never | 135 | 7342 | 1 | |
| | Ex | 6 | 667 | 0.59 | 0.26–1.34 |
| | Current | 13 | 1429 | 0.71 | 0.41–1.22 |
| Height quartile | <156 | 38 | 2493 | 1 | |
| | 156–159 | 39 | 2246 | 1.25 | 0.80–1.97 |
| | 160–163 | 36 | 2224 | 1.29 | 0.80–2.06 |
| | ≥164 | 44 | 2497 | 1.57 | 0.98–2.51 |
| Alcohol use | 0 | 102 | 5695 | 1 | |
| | 1–249 | 39 | 3112 | 0.85 | 0.58–1.26 |
| | ≥250 | 16 | 627 | 1.99 | 1.14–3.47 |
| Screening positive to bacteriuria | No | 138 | 8329 | 1 | |
| | Yes | 18 | 954 | 1.12 | 0.68–1.83 |
| Antibacterial treatment | No | 79 | 5369 | 1 | |
| | Yes | 78 | 4092 | 1.34 | 0.98–1.83 |

[a]Age-adjusted.

© 2000 Cancer Research Campaign

**Table 2** Relative risk[a] (95% confidence interval) of breast cancer between women with history of antibacterial treatment for bacteriuria and other women in categories of baseline screening for bacteriuria

| Baseline screening for bacteriuria | History of antibacterial medication for urinary tract infections | | | | | | | |
|---|---|---|---|---|---|---|---|---|
| | No | | | | Yes | | | |
| | No. at risk | No. of cases | RR | 95% CI | No. at risk | No. of cases | RR | 95% CI |
| Negative | 4811 | 70 | 1 | – | 3518 | 68 | 1.34 | 0.96–1.87 |
| Positive | 456 | 8 | 1.18 | 0.57–2.45 | 498 | 10 | 1.37 | 0.71–2.67 |

[a]Age-adjusted.

**Table 3** Relative risk[a] (95% confidence interval) of breast cancer between women with history of antibacterial treatment for bacteriuria and other women, by age and length of follow-up

| Follow-up (years) | Age (years) 0–49 (5536)[b] | | | 50–99 (3654)[b] | | | Total (9190)[b] | | |
|---|---|---|---|---|---|---|---|---|---|
| | No. of cases | RR | 95% CI | No. of cases | RR | 95% CI | No. of cases | RR | 95% CI |
| 1–10 | 33 | 1.47 | 0.73–2.97 | 37 | 1.14 | 0.59–2.22 | 70 | 1.24 | 0.77–1.99 |
| 11–18 | 53 | 1.93 | 1.11–3.37 | 32 | 0.78 | 0.37–1.67 | 85 | 1.37 | 0.89–2.12 |
| Total | 86 | 1.74 | 1.13–2.68 | 69 | 0.97 | 0.59–1.58 | 155 | 1.31 | 0.95–1.81 |

[a]Adjusted for age, region type, education, marital status, body mass index, parity, smoking, height, alcohol use and screening positive for bacteriuria.
[b]Women at risk.

cross-product of antibacterial treatment for bacteriuria and screening positive for bacteriuria confirmed this observation (Table 2). Those women previously treated for urinary tract infection and accordingly having no bacteriuria at baseline showed a RR of 1.35 (95% CI 0.96–1.87), whereas untreated women with newly diagnosed bacteriuria showed a RR of 1.18 (95% CI 0.57–2.45).

The associations between history of antibacterial treatment and breast cancer incidence varied considerably among age-groups. They were strongest in women under 40 years of age, were continuously reduced as a function of age and were lowest among women aged 60 years or more. The RR (history of antibacterial treatment positive vs negative) for the age-groups < 40 years, 40–49 years, 50–59 years and 60–69 years were 1.92 (95% CI 1.05–3.50), 1.46 (95% CI 0.80–2.66), 1.25 (95% CI 0.59–2.66) and 0.87 (95% CI 0.47–1.59) respectively.

With few exceptions, only weak associations existed between the history of antibacterial treatment for urinary tract infection and known potential risk factors of breast cancer, i.e. regional type, educational level, marital status, body mass index, height, parity and alcohol use. The strongest association was observed with parity, with a considerably low prevalence of antibacterial treatment among nulliparous women (data not shown). Accordingly, the relative risk for breast cancer among women who had received antibacterial treatment for urinary tract infection in comparison with those not receiving treatment was not notably altered by further adjustment for these factors (Table 3). The RR was 1.31 (95% CI 0.95–1.81) in all age-groups and 1.74 (95% CI 1.13–2.68) in women under 50 years of age.

The association between history of antibacterial treatment and breast cancer incidence was dependent on the length of follow-up (Table 3). It was strongest among women under 50 years of age with follow-up times of over 10 years; the RR was 1.93 (95% CI 1.11–3.37). No significant association was observed during the first 10 years of follow-up.

### DISCUSSION

The results of the present study suggest that premenopausal women using antibacterial treatment for bacteriuria have an elevated risk of developing breast cancer. A 74% excess risk was observed among women under 50 years of age, whereas no excess risk was noted among older women. The highest excess risk, 93%, was observed after a 10-year follow-up. These findings suggest that those women developing breast cancer are exposed to antibacterial medication at a relatively young age and that development of the disease requires more than 10 years. The follow-up period needed to demonstrate the association was relatively long. Since the long follow-up tends to weaken the representativeness of data on the antibacterial medication due to changes over time in use and type of antibiotics and chemotherapeutics, the excess risk found may be a conservative estimate. Furthermore, antibiotics are also commonly used for other reasons, which reduces contrast between the groups.

At the time of baseline examination, bacteriuria was considered potentially deleterious for kidneys, dysuria was regarded as a serious symptom and long period of medication followed by subsequent checkup were generally recommended. In the present study the history of antibacterial medication was based on questions about previous infections of the urinary tract, whereas data on neither the amounts of specific medication used nor on duration of use were available. These caveats, however, probably result in conservative estimates of the relative risks. The information on treatment for bacteriuria appeared to be valid since in the subsample of women with ongoing treatment, 97% reported the use of sulphonamides or combinations of such medications that were usually prescribed for bacteriuria at that time in Finland. The information on previous medication was also reliable since its repeatability after 6 months was relatively good. Although no meaningful hypothesis exists (Barnett and Stephens, 1997), the possibility cannot be fully excluded that the infection or some of

© 2000 Cancer Research Campaign its risk factors rather than the antibacterial treatment is the risk factor. The finding that baseline bacteriuria per se was not associated with subsequent breast cancer incidence, however, conflicts with that suggestion.

In accordance with findings from several previous studies (Harris et al, 1992), we found an elevated risk of breast cancer among women living in urban or industrial areas, having a higher level of education, with low parity, low body mass index and higher stature and among women using alcohol. It is thus plausible to expect that our finding of an independently elevated risk of breast cancer among young women who have received antibacterial treatment is also reliable. Since family disposition to breast cancer, age at menarche, age at birth of first child, age at menopause, benign breast disease, oral contraceptive use and post-menopausal oestrogen-replacement therapy, all of which are established or probable risk factors for breast cancer (Hulten et al, 1998), were not available in the present study, the association may be due to uncontrolled confounding. Dietary variables were also not available. On the other hand, it is reasonable to hypothesize that none of the risk factors mentioned are associated with bacteriuria or its treatment, and thus the antibacterial treatment/breast cancer relation may not be affected by adjustment for these factors.

In summary, we found an elevated risk of breast cancer in women who had received antibacterial treatment for urinary tract infection at a premenopausal age. This provides indirect suggestive evidence on the role of low lignan status in breast cancer development. Although our finding is biologically plausible and was independent of other risk factors for breast cancer available, the potential importance of uncontrolled factors in the relation cannot be excluded. Results from further observational epidemiological studies are needed before serious suspicions can be proposed about the involvement of antibiotics or chemotherapeutics in the aetiology of breast cancer.

### REFERENCES

Adlercreutz H (1998) Human health and phytoestrogens. In: *Reproductive and Development Toxicology*, Korach KD (ed), pp. 299–371. Marcel Dekker: New York

Adlercreutz H and Mazur W (1997) Phyto-oestrogens and Western diseases. *Ann Med* **29**: 95–120

Adlercreutz H, Fotsis T, Bannwart C, Wähälä K, Mäkelä T, Brunow G and Gorbach SL (1986) Determination of urinary lignans and phytoestrogen metabolites, potential antiestrogens and anticarcinogens, in urine of women on various habitual diets. *J Steroid Biochem* **25**: 791–797

Adlercreutz H, Fotsis T, Heikkinen R, Dwyer JT, Woods M, Goldin BR and Gorbach SL (1982) Excretion of the lignans enterolactone and enterodiol and of equol in omnivorous and vegetarian postmenopausal women and in women with breast cancer. *Lancet* **ii**: 1295–1299

Barnett BJ and Stephens DS (1997) Urinary tract infection: an interview. *Am J Med Sci* **314**: 245–249

Borriello SP, Setchell KD, Axelson M and Lawson AM (1985) Production and metabolism of lignans by the human faecal flora. *J Appl Bacteriol* **58**: 37–43

Cox DR (1972) Regression models and life-tables (with discussion). *J R Stat Soc B* **34**: 187–220

Harris JR, Lippman ME, Veronesi U and Willett W (1992) Breast cancer. *N Engl J Med* **327**: 319–328

Hulten K, Winkwist A, Adlercreutz H, Lenner P, Johansson R and Hallmans G (1998) A prospective study on lignans and breast cancer. The Cost 916 Workshop on Phyto-oestrogens: exposure, bioavailability, health benefits and safety concerns. Doorwerth, The Netherlands, 17–18 April

Ingram D, Sanders K, Kolybaba M and Lopez D (1998) Case–control study of phyto-oestrogens and breast cancer. *Lancet* **350**: 990–994

Knekt P, Marniemi J, Teppo L, Heliövaara M and Aromaa A (1998) Is low selenium status a risk factor for lung cancer? *Am J Epidemiol* **148**: 975–982

Setchell KD, Lawson AM, Borriello SP, Harkness R, Gordin H, Morgan DM, Kirk DN, Adlercreutz H, Anderson LC and Axelson M (1981) Lignan formation in man–microbial involvement and possible roles in relation to cancer. *Lancet* **ii**: 4–7

Teppo L, Pukkala E and Lehtonen M (1994) Data quality and quality control of a population-based cancer registry. Experiences in Finland. *Acta Oncol* **33**: 365–369

World Health Organization (1955) *International Classification of Diseases. Manual of international statistical classification of diseases, injuries, and causes of death, 7th Revision*. World Health Organization: Geneva

*© 2000 Cancer Research Campaign*
